# Osteotomy-assisted surgical crown lengthening combined with edgewise orthodontics for deep bite and gummy smile correction: a case report

**DOI:** 10.1097/RC9.0000000000000342

**Published:** 2026-03-20

**Authors:** Rifqi Atul Inayah, Tegar Arviga, Dik Megaputri Handayani, Ananto Ali Alhasyimi, Sri Suparwitri, Aulia Ayub

**Affiliations:** a Orthodontics Residents, Faculty of Dentistry, Universitas Gadjah Mada, Yogyakarta, Indonesia; b Periodontics Residents, Faculty of Dentistry, Universitas Gadjah Mada, Yogyakarta, Indonesia; c Departement of Orthodontics, Faculty of Dentistry, Universitas Gadjah Mada, Yogyakarta, Indonesia

**Keywords:** bidental retroclination, cephalometric analysis, class I malocclusion, crowding, deep bite, orthodontic treatment

## Abstract

**Introduction:**

Deep bite is a vertical occlusal discrepancy characterized by excessive anterior overlap, which may compromise dental esthetics, function, and structural integrity. An interdisciplinary approach integrating orthodontic therapy with periodontal crown lengthening can enhance tooth proportion and improve smile harmony.

**Case Presentation:**

A 24-year-old woman presented to the Oral and Dental Hospital of Universitas Gadjah Mada (RSGM UGM Prof. Soedomo), Yogyakarta, with complaints of anterior crowding, protrusion, deep bite, and a gummy smile. Clinical examination revealed a normal facial profile, a 3.63-mm overjet, a 6.7-mm overbite, and a Class I molar and canine relationship. Cephalometric analysis (SNA 83.17°, SNB 80.55°, ANB 2.63°) confirmed a Class I dentoskeletal pattern with bidental retrusion and mandibular anterior crowding.

**Discussion:**

Orthodontic treatment using a Standard Multiloop appliance effectively corrected the deep bite and aligned both arches, achieving a final overjet of 2.4 mm and an overbite of 2.0 mm. Subsequent crown-lengthening performed by a periodontics resident at the RSGM UGM Prof. Soedomo further refined gingival contours and enhanced smile esthetics.

**Conclusion:**

The integration of multiloop orthodontic mechanics and periodontal crown lengthening represents a predictable and comprehensive interdisciplinary strategy for managing deep bite with a gummy smile, resulting in significant improvements in occlusion, tooth proportions, and facial esthetics.

## Introduction

Deep bite and excessive gingival display are complex dentoalveolar and periodontal conditions that often require a multidisciplinary approach integrating orthodontic biomechanics, periodontal surgery, and facial esthetic considerations. Successful deep bite correction relies on the evaluation of vertical discrepancies, occlusal function, skeletal proportions, and long-term periodontal stability. In cases accompanied by vertical maxillary excess or anterior dentoalveolar extrusion, maxillary or mandibular osteotomy may be considered to achieve stable vertical correction^[^1,2^]^.

Altered passive eruption (APE) is one of the most common intraoral causes of a gummy smile. In APE Type I, subtype B, the alveolar crest is positioned very close to the cemento-enamel junction (CEJ), resulting in short clinical crowns due to incomplete passive eruption. Unlike Subtype A, which can be treated with gingivectomy alone, Subtype B requires crown lengthening with osseous reduction (osteotomy or ostectomy) to reestablish the biologic width and achieve stable gingival contours^[^2–4^]^. Non-surgical approaches are inadequate because the underlying bone remains the primary determinant of the excessive gingival display. Other causes of a gummy smile include gingival hyperplasia, compensatory eruption due to attrition, anterior dentoalveolar extrusion, vertical maxillary excess (VME), a short upper lip, and upper lip hypermobility. Treatment selection should be based on the underlying etiology: crown lengthening for APE, orthodontic treatment or lip repositioning for mild-to-moderate VME, orthognathic surgery for severe VME, and lip repositioning or botulinum toxin injections for lip hypermobility^[^5–7^]^.

HIGHLIGHTS

- Managing a severe deep bite case with short tooth crowns using Multiloop Edgewise presents a clinical challenge. Surgical and orthodontic interventions have the potential to comprehensively resolve this case.
- The surgical treatment with crown lengthening resulted in a successful and rapid recovery, without the development of hypertrophic scarring.
- Excellent treatment planning contributed to an acceptable, aesthetic, structural, and functional outcome.

Recent developments in orthodontic biomechanics highlight the advantages of the Multi-Loop Edgewise Archwire (MEAW) system in achieving controlled three-dimensional tooth movement. Through light, continuous forces, MEAW enables precise anterior intrusion, reliable torque modulation, and coordinated adjustment of the occlusal plane. These characteristics make MEAW particularly valuable in cases presenting vertical discrepancies^[^2,6^]^. Contemporary clinical reports indicate that integrating MEAW mechanics with temporary anchorage devices can further enhance vertical stability by reinforcing posterior anchorage and minimizing undesirable dental side effects. This combined approach has been shown to improve the predictability of vertical correction, limit relapse, and maintain occlusal plane orientation more effectively than conventional methods. Evidence from recent case reports involving complex malocclusions including skeletal Class III patterns and open-bite conditions demonstrates that MEAW–TAD protocols support more efficient biomechanical control and promote stable long-term outcomes^[^4,8^]^

Therefore, the combination of MEAW mechanics, crown lengthening, and osseous surgery provides a comprehensive and predictable approach for managing deep bite associated with a gummy smile. This case report is presented in accordance with the SCARE 2025 guidelines^[^9^]^.

## Case presentation

A 24-year-old woman presented to the Universitas Gadjah Mada Oral and Dental Hospital (RSGM UGM Prof. Soedomo), Yogyakarta, reporting chief concerns of anterior crowding, dental protrusion, deep bite, and excessive gingival display when smiling. The patient was medically healthy with no notable systemic history.

**Figure 1.**
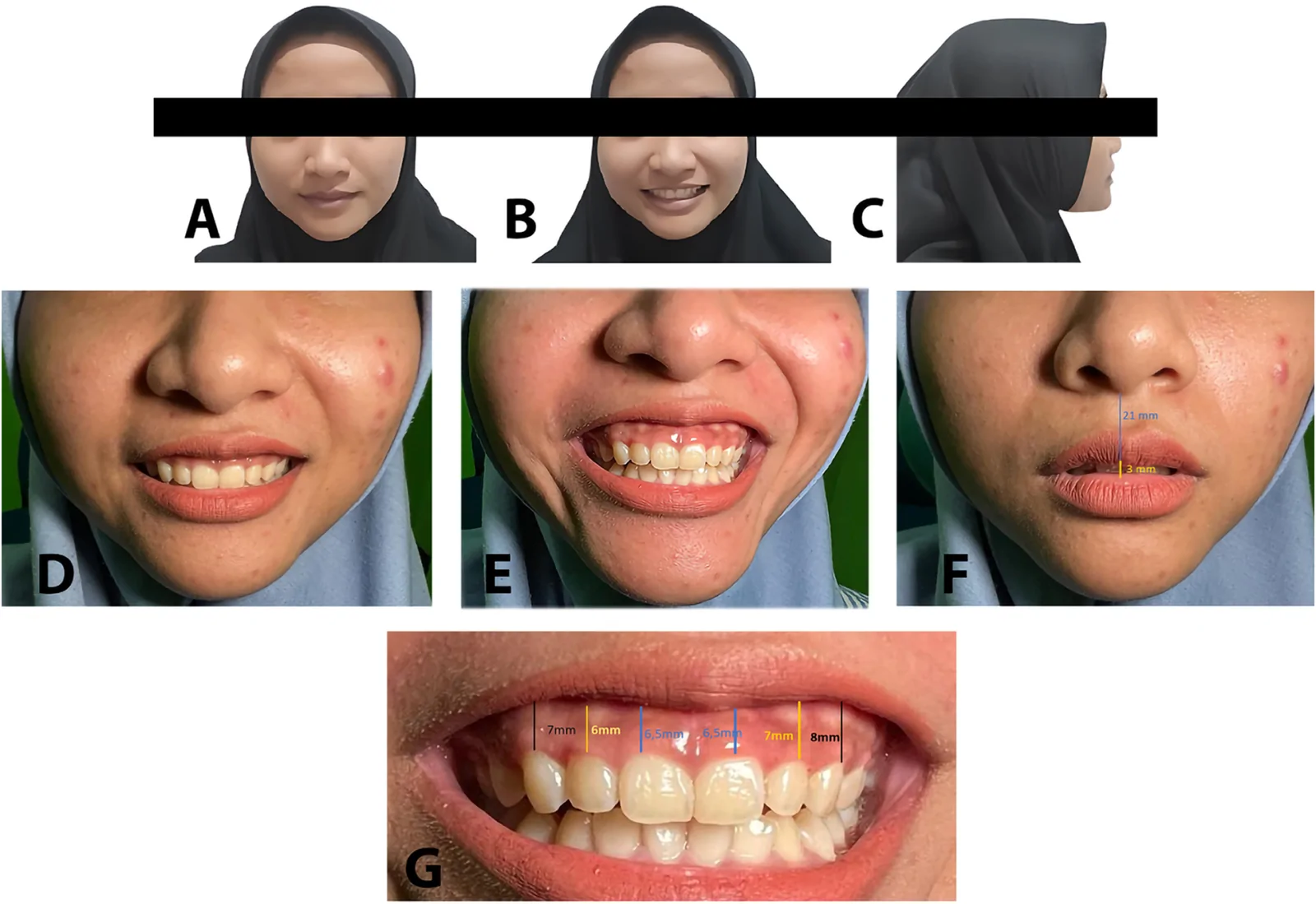
Pre-treatment extraoral and intraoral photographs of the patient. (A) Frontal view at rest. (B) Frontal smiling view. (C) Lateral profile view. (D) Close-up view of the anterior teeth at rest. (E) Close-up view of the smile showing excessive gingival display. (F) Measurement of upper lip length and incisal display at rest. (G) Clinical crown height measurements of the maxillary anterior and premolar teeth.

Extraoral examination revealed a symmetrical mesoprosopic facial form and a mildly convex profile. Resting lip measurements demonstrated a subnasale-to-upper lip distance of 21 mm and an incisal display of 3 mm, effectively ruling out a short upper lip. Speech and swallowing functions were within normal limits. Cephalometric parameters were also within the normative range, thereby excluding vertical maxillary excess as a contributing factor. Based on these findings, upper lip hypermobility was identified as the primary etiologic factor associated with the patient’s excessive gingival display (Fig. 1).

**Figure 2.**
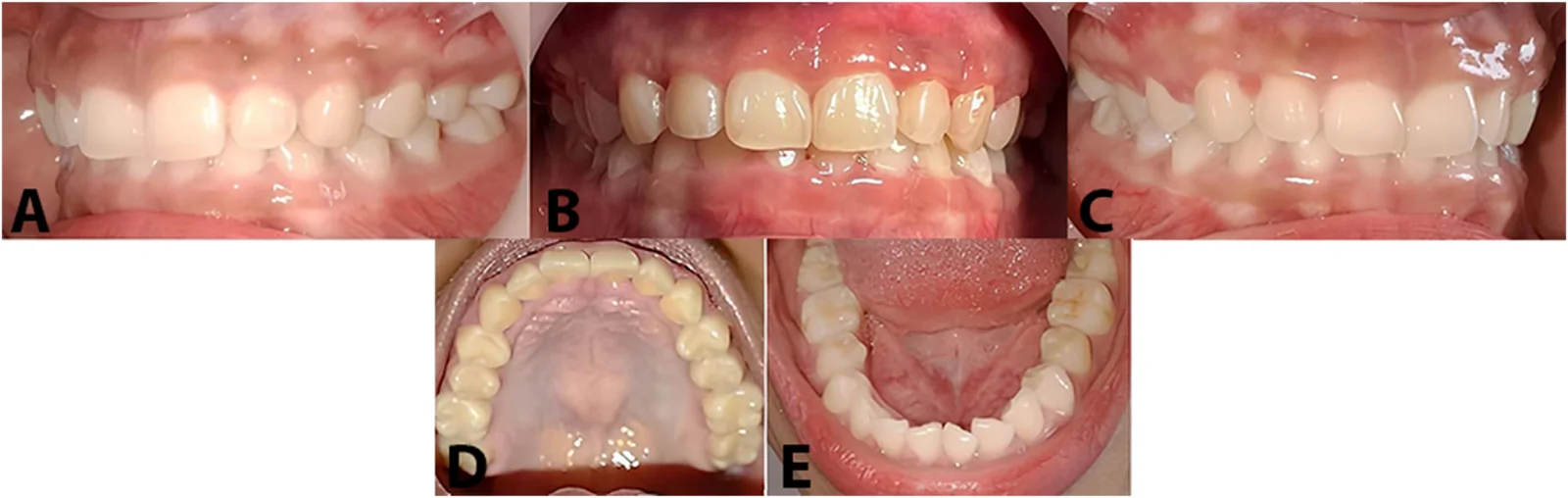
Pre-treatment intraoral photographs showing dental and occlusal relationships. (A) Right buccal view in maximum intercuspation. (B) Frontal occlusion view demonstrating deep anterior overbite. (C) Left buccal view in maximum intercuspation. (D) Maxillary occlusal view showing arch form and anterior crowding. (E) Mandibular occlusal view demonstrating mild anterior crowding.

Intraoral assessment showed more than 5 mm of gingival exposure upon smiling (Fig. 2). The width of keratinized gingiva exceeded 5 mm, and the patient demonstrated good oral hygiene with no signs of inflammation. Gingival health was confirmed by a Löe–Silness Gingival Index score of 0. Oral hygiene instructions were reinforced, emphasizing the Modified Bass brushing technique. Occlusal evaluation indicated an overjet of 3.63 mm and an overbite of 6.7 mm, with bilateral Angle Class I molar and canine relationships. The maxillary and mandibular midlines were coincident, and the deep bite was primarily attributed to anterior dental supracclusion.

**Figure 3.**
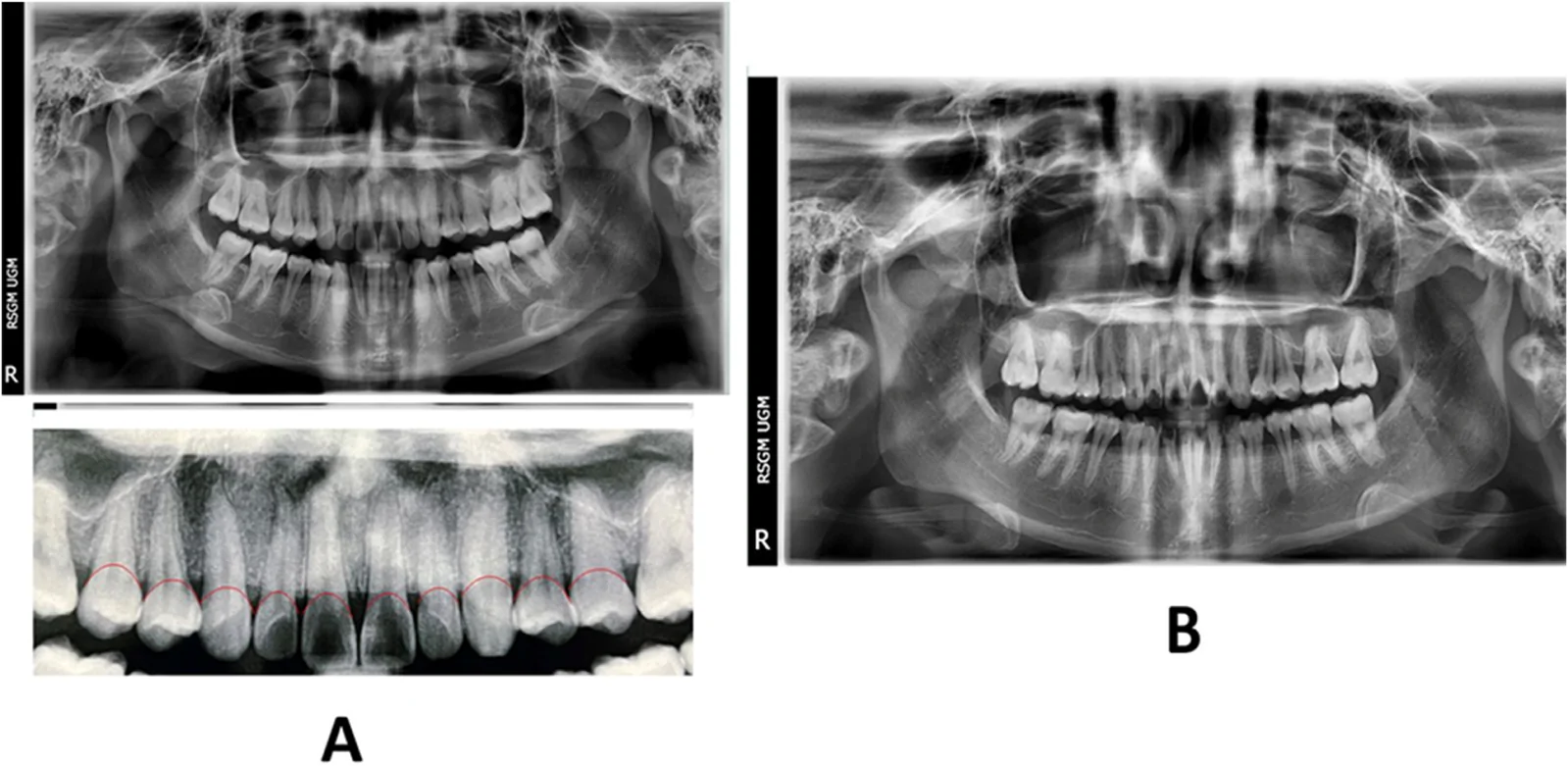
Pre- and post-treatment radiographic evaluation. (A) Pre-treatment panoramic radiograph showing overall dentition and alveolar bone morphology. The periapical radiograph illustrates the cemento-enamel junction (CEJ) contour from teeth 15–25, highlighted with a red guideline to demonstrate the coronal position of the alveolar crest relative to the CEJ. (B) Post-treatment panoramic radiograph demonstrating improved overall dental alignment following orthodontic therapy and periodontal crown lengthening.

Bone sounding (Table 1) and pre-treatment panoramic radiography (Fig. 3) revealed the alveolar crest positioned at or slightly coronal to the CEJ. This reduced biological width was consistent with a diagnosis of APE Type I Subtype B affecting teeth 15–25, indicating the need for a periodontal intervention involving osseous modification.

**Table 1:** Clinical measurements, probing depth, bone sounding, and planned osseous reduction (teeth 15–25).

Tooth	Clinical crown height (mm)	Clinical crown width (mm)	Ideal height (mm)	Probing depth (M/B/D, mm)	Bone sounding (M/B/D, mm)	Gingivectomy (mm)	Planned osseous reduction (mm)
15	6.0	7.0	8.5	3/2/2	4/3/1	2.5	2.5
14	6.5	7.0	8.5	2/1/2	4/2/3.5	2.0	3.0
13	7.0	7.5	9.0	2/1/2	4/3/4	2.0	2–3
12	5.5	6.0	7.5	2/2/2	3.5/3/3	2.0	2–3
11	6.5	7.0	8.5	3/2/2	3.5/3/3	2.0	2–3
21	6.5	7.0	8.5	3/2/2	4/3/3	2.0	2–3
22	5.5	6.0	7.5	2/2/3	2/2/3	2.0	2–3
23	7.0	7.5	9.0	2.5/2.5/4	4/4/5	2.0	1–2
24	6.5	7.5	9.0	2/2/2	4/3/3	2.5	2.5
25	5.5	7.0	8.5	3/2.5/3	4/4/4	3.0	2.0

**Figure 4.**
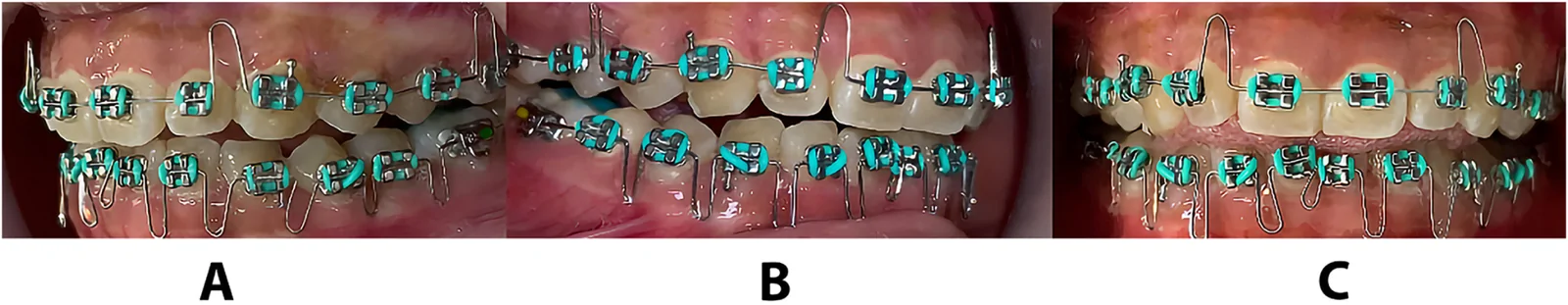
Intraoral photographs demonstrating the use of Multiloop Edgewise Archwire (MEAW) mechanics during the active orthodontic phase. (A) Right lateral view showing anterior bite turbos and MEAW loops designed for vertical control and anterior intrusion. (B) Left lateral view illustrating coordinated MEAW activation for leveling and occlusal plane adjustment. (C) Frontal view displaying full appliance engagement with upper and lower MEAW wires to achieve deep bite correction.

Orthodontic treatment was carried out over a 24-month period using a 0.022” standard edgewise appliance system (Ormco, USA). During the initial phase (months 0–3), leveling and alignment were performed with 0.016” stainless steel archwires, assisted by vertical loops for anterior alignment. Bite turbos were placed on teeth 11 and 21 to facilitate bite opening and enable bracket bonding on the mandibular anterior teeth. In the subsequent phase (months 4–8), a 0.016 × 0.022” stainless steel archwire was used to correct malposition and resolve crowding. Deep-bite correction was achieved between months 9 and 14 using a 0.016 × 0.022” MEAW archwire, providing vertical control, anterior intrusion, and occlusal plane modification. Torque and finishing adjustments were performed from months 15 to 20, followed by monitoring of pre-retention stability from months 21 to 24 prior to appliance removal and placement of fixed retainers in both arches (Fig. 4).

**Figure 5.**
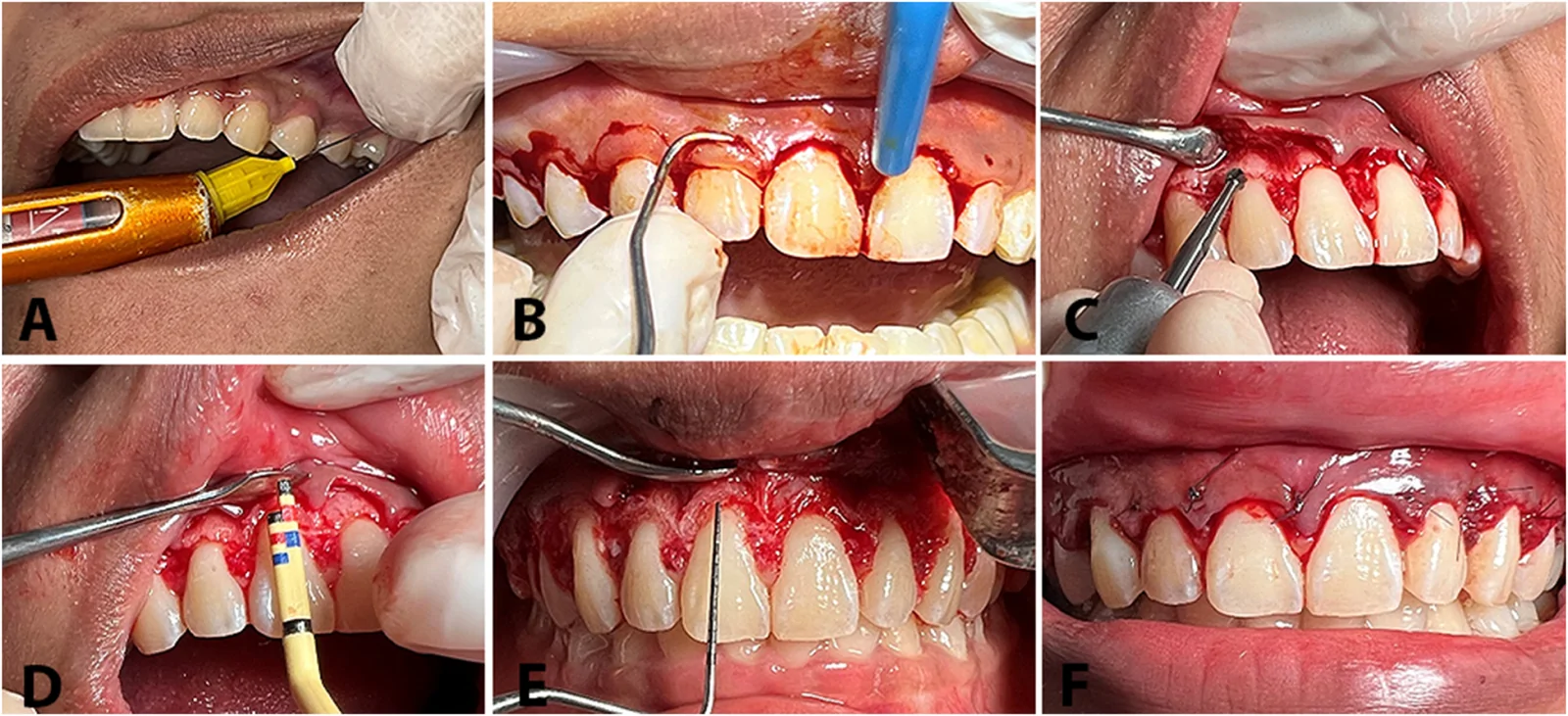
Sequential clinical photographs illustrating the esthetic crown lengthening procedure.(A) Local anesthesia administration prior to the surgical procedure. (B) External bevel gingivectomy performed to expose the underlying tissue and establish the desired gingival margin contour. (C) Full-thickness flap elevation revealing the position of the alveolar crest. (D) Bone sounding and measurement of the biologic width to determine the extent of osseous recontouring required. (E) Osseous reduction (osteotomy and osteoplasty) performed to re-establish adequate biologic width and harmonize the gingival architecture. (F) Flap repositioning and suturing to achieve stable gingival margins following osseous modification.

After completion of orthodontic alignment, esthetic crown lengthening with ostectomy was performed on teeth 15–25 to manage APE Type I, subtype B. The procedure began with povidone-iodine asepsis and infiltration anesthesia (2% lidocaine with 1:100,000 epinephrine). Gingivectomy was carried out with Chu Gauge guidance, followed by full-thickness flap elevation via intrasulcular incisions. Ostectomy and osteoplasty were performed to re-establish a minimum CEJ–crest distance of ≥3 mm. Scaling and root planing were then completed prior to closure with 5-0 nylon sutures, and Reso-pac was applied. Postoperative care included amoxicillin 500 mg, dexamethasone 0.5 mg, and mefenamic acid 500 mg every 8 hours, along with hyaluronic acid gel and 0.2% chlorhexidine mouth rinse (Fig. 5).

**Figure 6.**
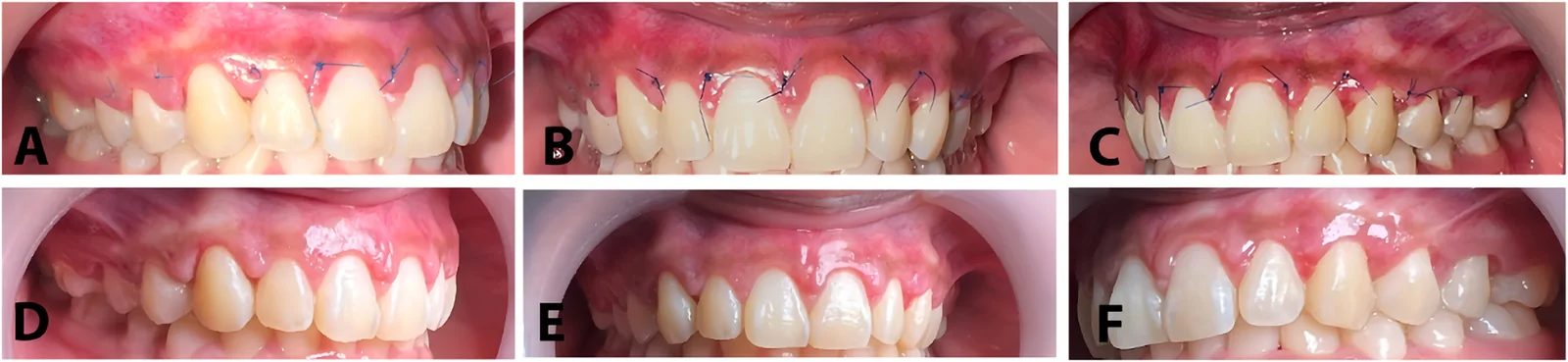
Postoperative soft-tissue healing after esthetic crown lengthening with osseous recontouring. (A–C) One-week postoperative control, showing mild gingival inflammation, early epithelialization, and initial stabilization of the repositioned flap. (D–F) Three-month postoperative evaluation, demonstrating complete resolution of inflammation, well-contoured gingival architecture, and stable gingival margin positioning with improved clinical crown proportions.

Short-term follow-up (1 week–3 months) demonstrated favorable soft-tissue healing without complications, characterized by stable gingival margins and the absence of inflammatory signs. These clinical findings were consistent with the quantitative measurements of gingival height, which revealed a reduction in the distance from the inferior vermilion border of the upper lip to the gingival margin following crown lengthening. As shown in Table 2, an average reduction of approximately 2 mm was observed across the maxillary anterior region (teeth 13–23), indicating effective correction of excessive gingival display. At the 6-month evaluation, gingival contours remained stable with no recurrence of excessive gingival display; the overbite measured 2.0 mm and the overjet 2.4 mm. At the 12-month follow-up, no relapse of the gummy smile was observed, deep-bite correction remained stable, and further improvement in facial soft-tissue harmony was noted, with no clinical signs or symptoms of temporomandibular disorder (TMD) (Fig. 6).

**Table 2:** Vertical height measurements from the inferior vermilion of the upper lip to the gingival margin for teeth 13–23 before and after crown lengthening (CL).

Height (distance from inferior vermilion of the upper lip to the gingival margin)	Tooth element					
	13	12	11	21	22	23
Before CL (mm)	7	6	6.5	6.5	7	8
After CL (mm)	5	4	4.5	4.5	5	6

**Figure 7.**
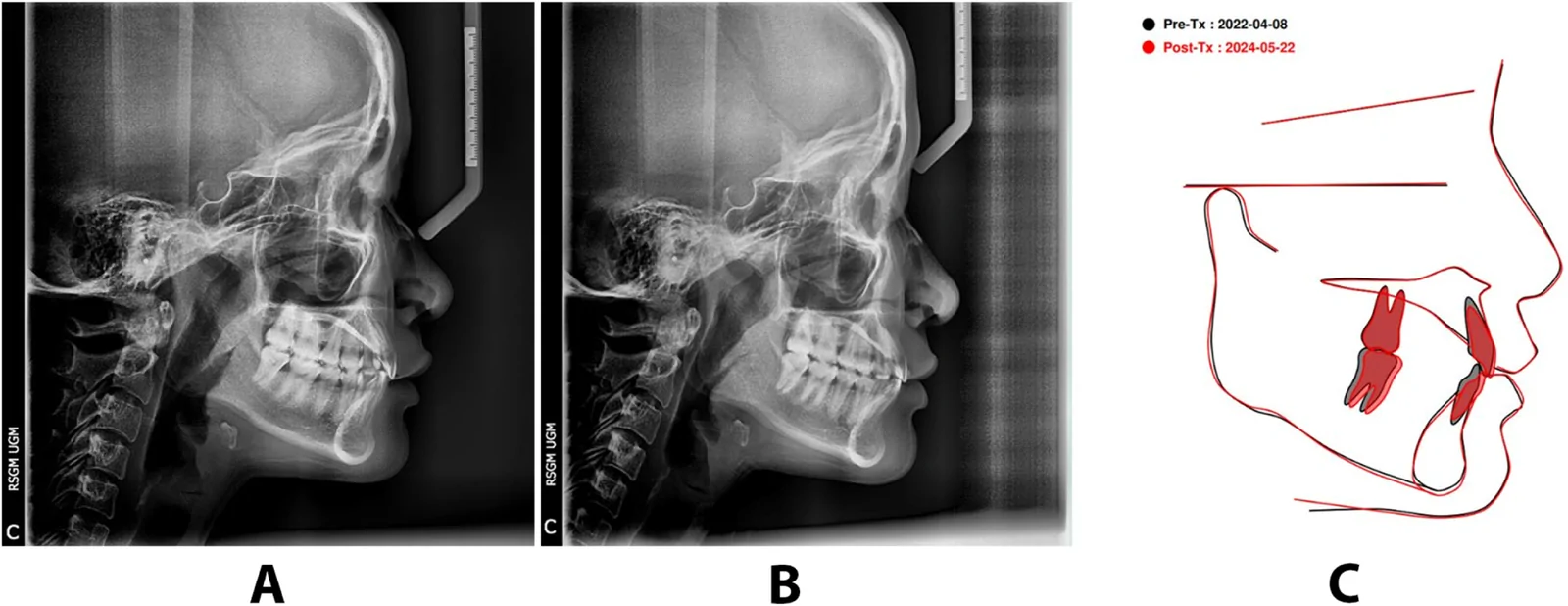
Lateral cephalometric evaluation and Superimposition. (A) Pre-treatment lateral cephalometric radiograph, showing initial skeletal and dentoalveolar relationships prior to orthodontic intervention. (B) Post-treatment lateral cephalometric radiograph, demonstrating improved incisor inclination, overbite correction, and stabilization of the occlusal plane following MEAW mechanics. (C) Cephalometric superimposition (pre-treatment in black, post-treatment in red) illustrating anterior intrusion, controlled incisor torque, and vertical changes achieved during orthodontic therapy.

Post-treatment cephalometric evaluation using Downs and Steiner analyses, compared with normative values, demonstrated favorable skeletal and dental changes. SNA increased from 83.17° to 83.26° (normal 82° ± 2°), SNB from 80.55° to 80.71° (normal 80° ± 2°), and ANB from 2.63° to 2.80° (normal 2° ± 2°), confirming a stable skeletal Class I pattern. Maxillary incisor inclination increased from 103° to 108° (normal 109° ± 6°) and mandibular incisor inclination from 88.14° to 95.90° (normal 90° ± 5°) (Table 3). Cephalometric superimposition demonstrated favorable dentoalveolar movements and improved soft-tissue profile, consistent with enhanced occlusal and esthetic outcomes (Fig. 7).

**Table 3:** Lateral cephalometric measurements before (pre-treatment) and after treatment (post-treatment).

Parameters	Normal (mean ± SD)	Pre-treatment	Post-treatment
Horizontal skeletal			
SNA (^o^)	82 ± 2	83.17	83.26
SNB (^o^)	80 ± 2	80.55	80.71
ANB (^o^)	2 ± 2	2.63	2.55
Angle of convexity (^o^)	0 ± 5	4.48	5.59
Vertical skeletal			
Y-axis (^o^)	60 ± 4	61.41	62.69
Dental			
Interincisal angle (^o^)	135 ± 10	143.07	129.07
U1-palatal plane (^o^)	109 ± 6	103	108
U1-NA (mm)	4 ± 2	1.23	3.19
L1-NB (mm)	4 ± 2	2.69	6.27

ANB, A point–nasion–B point angle; SNA, sella–nasion–A point angle; SNB, sella–nasion–B point angle.

**Figure 8.**
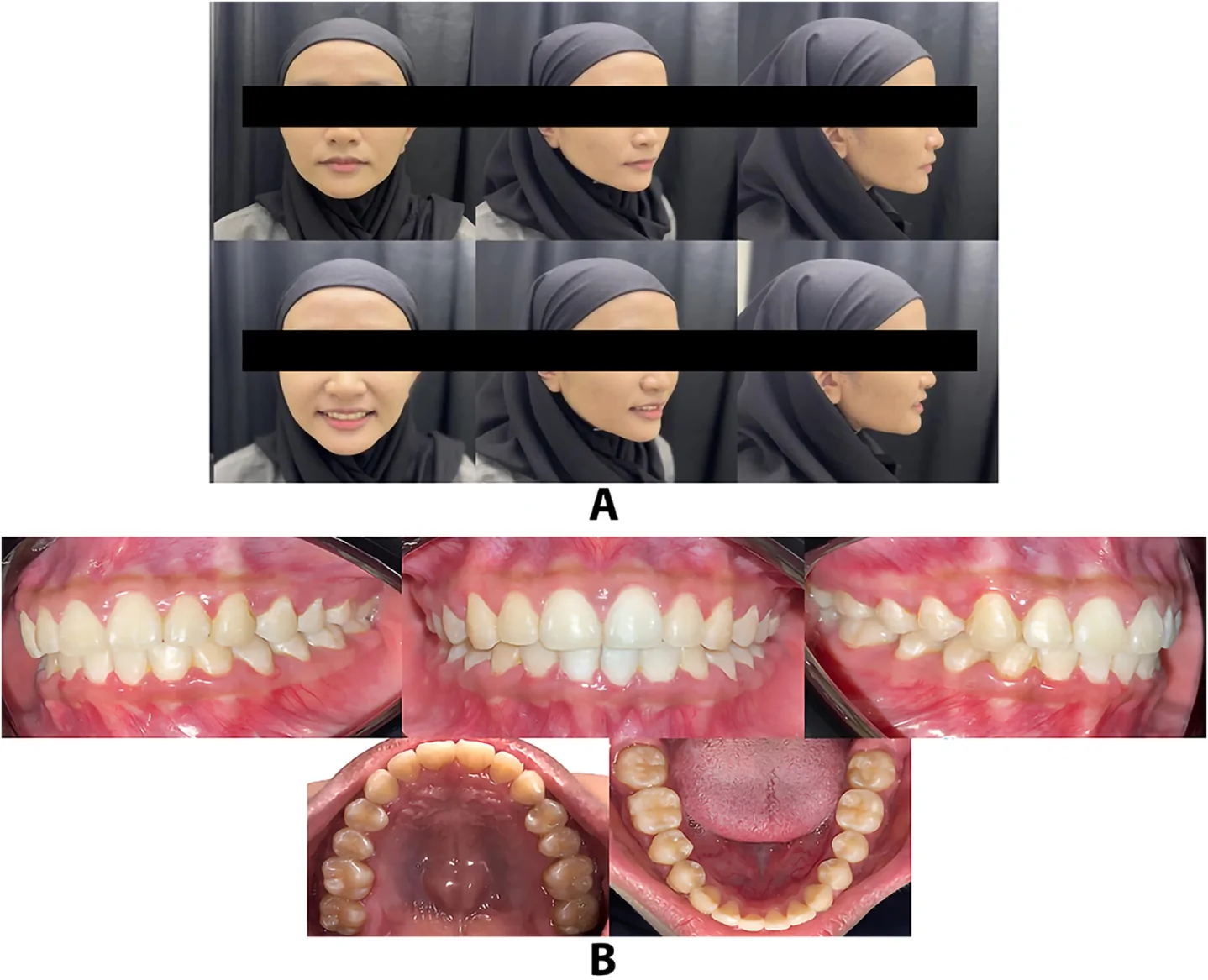
Final extraoral and intraoral photographs after orthodontic treatment and crown lengthening. (A) Post-treatment extraoral photographs showing improved facial aesthetics, balanced smile line, and harmonious soft-tissue profile following completion of orthodontic therapy and esthetic crown lengthening. (B) Post-treatment intraoral photographs demonstrating stable occlusion, corrected overbite and overjet, coordinated dental arches, and appropriate gingival contour following osseous crown lengthening.

Overall, the combination of orthodontic therapy and bone-reduction crown lengthening effectively corrected the overjet from 3.63 mm to 2.4 mm, reduced the deep-overbite from 6.7 mm to 2 mm, and resolved mild crowding in both arches. These improvements resulted in a more harmonious smile and a better-defined soft-tissue facial profile (Fig. 8).

## Discussion

In this case, the combination of orthodontic treatment using Multiloop Edgewise Archwire (MEAW) mechanics and esthetic crown lengthening with osseous recontouring served as the primary approach for managing a severe deep bite associated with APE and excessive gingival display^[^10,11^]^. During the initial orthodontic phase, the use of anterior bite turbos and stainless-steel archwires with vertical loops facilitated stable leveling and alignment. Once alignment was achieved, MEAW mechanics provided refined control over anterior intrusion, occlusal plane adjustment, and incisor torque. The clinical improvements – including the reduction of overbite from 6.7 mm to 2.0 mm and the decrease in overjet from 3.63 mm to 2.4 mm – highlight the effectiveness of MEAW as a non-extraction strategy for complex vertical discrepancies^[^7,12,13^]^.

Several alternative methods for anterior intrusion have been described, including the Burstone intrusion arch, Ricketts utility arch, posterior bite-block appliances, aligners with bite ramps, and temporary anchorage device (TAD)-supported intrusion. Although these techniques may yield satisfactory intrusion, they have inherent limitations such as a tendency toward incisor proclination, less predictable torque control, or a high dependency on patient compliance. In comparison, MEAW enables more comprehensive three-dimensional control of anterior tooth position and molar extrusion, making it advantageous in cases presenting simultaneous vertical problems and crowding^[^7,13,14^]^.

The excessive gingival display observed in this patient was primarily attributed to a combination of APE Type I Subtype B and upper lip hypermobility. Clinically, the gingival margins were positioned more coronally than ideal, producing short and square-shaped clinical crowns with an excessive band of keratinized gingiva, consistent with the phenotypic characteristics of APE^[^1,3^]^. Periodontal evaluation further confirmed this diagnosis through bone sounding, which revealed that the alveolar crest was positioned extremely close to the CEJ in several teeth, indicating an insufficient biological width. Quantitatively, reduced CEJ–crest distances were documented at multiple sites, including tooth 15 (1 mm distally), tooth 14 (2 mm buccally and 3.5 mm distally), tooth 22 (2 mm mesially and buccally), and similar deficiencies across teeth 13, 12, 11, 21, 23, 24, and 25. Among these, teeth 15, 14, and 22 exhibited biological width values of less than 2 mm, necessitating osseous crown lengthening to re-establish an appropriate biological width of at least 3 mm. These findings were corroborated by panoramic radiography, which demonstrated that the alveolar crest lay at or slightly coronal to the CEJ across the maxillary anterior region. Moreover, cephalometric analysis demonstrated values within normative limits, excluding vertical maxillary excess, while a subnasale–upper lip distance of 21 mm and normal incisal display at rest ruled out a short upper lip. Collectively, these clinical, periodontal, and radiographic assessments confirmed the diagnosis of APE Type IB combined with upper lip hypermobility as the principal etiologic factors contributing to the patient’s gummy smile^[^15^]^.

Crown lengthening with osseous reduction was indicated because gingivectomy alone would not provide long-term gingival margin stability in APE Type IB, where the alveolar crest is positioned too coronally^[^5,16^]^. Osteotomy and osteoplasty were necessary to re-establish a biological width of at least 2–3 mm, which is essential for preventing recurrent periodontal inflammation and maintaining a stable gingival margin. Bone sounding values at teeth 15, 14, and 22 demonstrated less than 2 mm of biological width, confirming the need for osseous modification. In other regions, osteoplasty was performed to harmonize the anterior gingival contour^[^17,18^]^.

Alternative therapies, such as orthodontic forced eruption, surgical extrusion, or esthetic lip repositioning, may be appropriate in select cases; however, they were not chosen here. Forced eruption is generally more suitable for isolated cases with diminished crown structure, whereas lip repositioning is typically reserved for patients with severe upper lip hypermobility. In this case, osseous crown lengthening alone provided sufficient esthetic correction without the need for adjunctive procedures^[^15,19,20^]^.

Periodontal monitoring demonstrated that the soft tissues were healthy prior to surgery, as indicated by a Löe–Silness Gingival Index score of 0. The patient was re-instructed in the Modified Bass brushing technique to optimize oral hygiene. One month after the procedure, mild inflammation was observed in the interdental areas of teeth 13 and 22–23, likely related to incomplete flap adaptation or insufficient bone reduction in those regions. These findings were successfully managed with curettage and gingivoplasty, with no adverse effect on long-term stability^[^5,16^]^.

The periodontal procedure did not affect the orthodontic biomechanics, as crown lengthening was performed only after all active orthodontic movements had been completed and appliances removed. Consequently, the orthodontic treatment plan did not require modification, tooth movement was neither accelerated nor delayed, and postoperative inflammation had no impact on orthodontic outcomes. The only considerations after surgery involved maintaining optimal oral hygiene and ensuring the fixed retainers were bonded without irritating the surgical sites^[^19,21^]^.

This case report has several limitations. The 12-month follow-up period limits conclusions regarding long-term periodontal and orthodontic stability. Quantitative periodontal parameters such as clinical attachment levels and standardized plaque indices were not recorded. In addition, cone-beam computed tomography was not used, restricting the ability to assess alveolar bone changes in three dimensions. As a single case report, these findings cannot be generalized without additional clinical research.

Overall, the findings of this case underscore the substantial clinical value of an integrated orthodontic–periodontal approach in the management of Class I skeletal patterns with severe deep bite, APE Type IB, and upper lip hypermobility. The combined use of MEAW mechanics and esthetic crown lengthening with osteotomy proved to be a conservative yet highly predictable strategy for correcting vertical discrepancies, re-establishing an adequate biologic width, and achieving stable gingival margin positioning. Clinically, this multidisciplinary protocol contributed meaningfully to improving crown proportions, enhancing gingival symmetry, and achieving a more harmonious and esthetically pleasing smile – all without resorting to more invasive procedures such as orthognathic surgery. The integration of orthodontic and periodontal expertise ensured long-term functional and esthetic stability, ultimately resulting in high patient satisfaction. These outcomes highlight the essential role of coordinated interdisciplinary care in managing complex dentoalveolar–periodontal conditions.

## Conclusion

The successful management of this case demonstrates that combining MEAW-based orthodontic biomechanics with esthetic crown lengthening is an effective and conservative approach for treating severe deep bite associated with APE Type IB and excessive gingival display. MEAW mechanics provided predictable vertical control and stable anterior intrusion, while crown lengthening with osseous recontouring restored appropriate biological width, improved crown proportions, and resulted in a more harmonious smile. Multidisciplinary collaboration between orthodontics and periodontics was essential in addressing the functional and esthetic components of this complex dentoalveolar condition. This integrated treatment approach yielded stable occlusal results, enhanced facial and dental esthetics, and high patient satisfaction at the 12-month follow-up.

## Data Availability

The data supporting the findings of this study are available within the article.
